# Deforming nodular lesions in an indigenous person from western Mexico

**DOI:** 10.1002/ski2.452

**Published:** 2024-08-31

**Authors:** Georgina Rodriguez Miramontes, Ossmara Cervantes Villegas, Dianely A. García‐Hernández, José Alberto García‐Lozano

**Affiliations:** ^1^ Universidad Autónoma de Nayarit Servicio de Medicina Interna Hospital de Especialidades “Dr. Antonio González Guevara” Tepic Nayarit Mexico; ^2^ Departamento de Introducción a la Clínica Facultad de Medicina y Hospital Universitario “Dr. José E. González’’ Universidad Autónoma de Nuevo León Monterrey Nuevo León Mexico

## Abstract

Deforming nodular lesions in an indigenous person from western Mexico compatible with leishmaniasis.

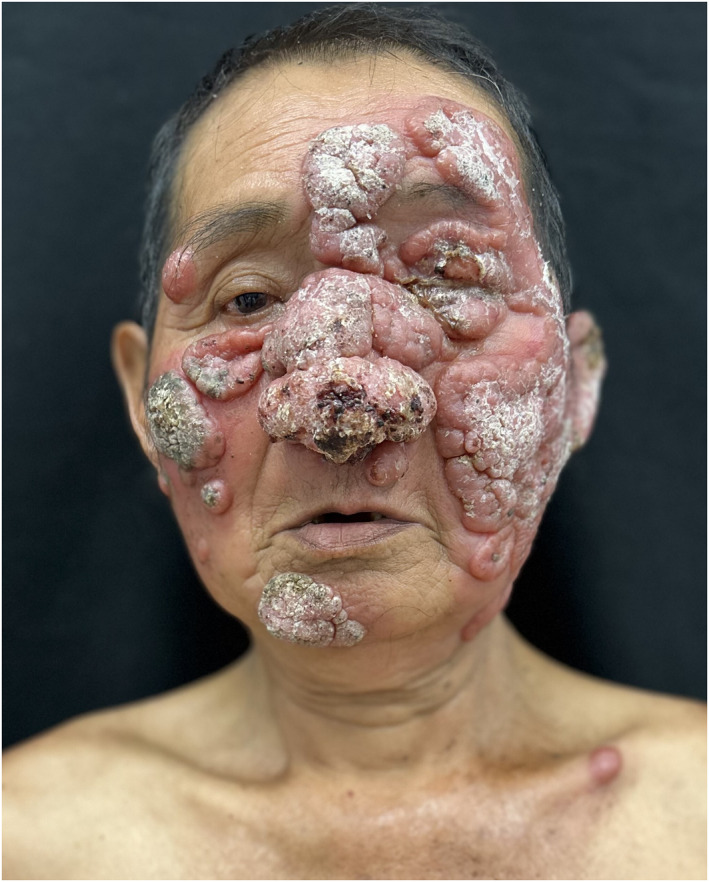

Dear Editor,

We present a 68‐year‐old immunocompetent man with widespread skin lesions involving the face, trunk, arms and legs that developed over a period of 2 years. He worked in a rural coffee plantation in Nayarit, Mexico (Figure 1a,b). On physical examination, indurated erythematous nodules and foul‐smelling ulcers were found.^1^ The diagnosis was compatible with leishmaniasis, confirmed by the visualisation of amastigotes in the cytological and histopathological analyses^1^ (Figure 1c,d). The patient was treated with liposomal amphotericin B at 3 mg/kg/day. Leishmaniasis is an underdiagnosed disease, endemic to tropical areas with transmission through anthroponotic and zoonotic cycles. Access to treatment is limited in some settings.^2^

**FIGURE 1:**
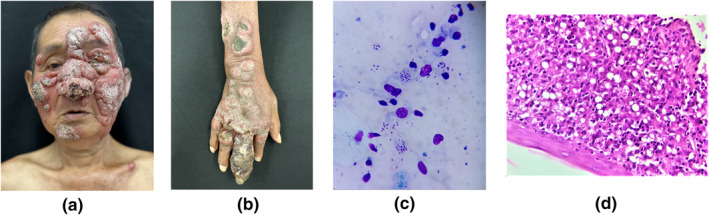
(a) Deforming nodular lesions on the face; (b) nodular lesions that disseminate to the trunk, arms and legs; (c) amastigotes in the cytological image and (d) amastigotes in the histopathological analyses.

## CONFLICT OF INTEREST STATEMENT

None to declare.

## AUTHOR CONTRIBUTIONS

**Georgina Rodriguez Miramontes**: Writing—original draft (equal). **Ossmara Cervantes Villegas**: Writing—original draft (equal). **Dianely A. García‐Hernández**: Formal analysis (equal); investigation (equal); methodology (equal); writing—review and editing (equal). **José Alberto García‐Lozano**: Project administration (equal); supervision (equal); validation (equal); visualisation (equal); writing—review and editing (equal).

## ETHICS STATEMENT

This study was approved by the authors' IRB.

## PATIENT CONSENT

Written patient consent for publication was obtained.

## Data Availability

Data are openly available in a public repository that issues datasets with DOIs.
